# Multidisciplinary Advanced Practice Nursing-Led Vascular Access Care Pathway in Patients with Cystic Fibrosis: A Practice-Based Descriptive Analysis

**DOI:** 10.3390/healthcare14152414

**Published:** 2026-08-05

**Authors:** Ana María Montserrat Gala, José Fernández-Sáez, Raquel Ayuso-Margañón, Amalia Sillero Sillero

**Affiliations:** 1 Department of Medicine, Universitat Autònoma de Barcelona (UAB), 08193 Barcelona, Spain; anamaria.montserrat@autonoma.cat; 2 Servei Atenció Primària Terres de l’Ebre, Institut Català de la Salut, 43500 Tortosa, Spain; 3 Unitat de Suport a la Recerca Terres de l’Ebre, Fundació Institut Universitari per a la Recerca a l’Atenció Primària de Salut Jordi Gol i Gurina (IDIAPJGol), 43500 Tortosa, Spain; 4 Facultat de Infermeria, Campus Terres de l’Ebre, Universitat Rovira i Virgili, 43500 Tortosa, Spain; 5 Social Determinants and Health Education Research Group (SDHEd), Hospital del Mar Research Institute, 08003 Barcelona, Spain; 6 Hospital del Mar Nursing School (ESIHMar), Universitat Pompeu Fabra-Affiliated, 08003 Barcelona, Spain; 7 Escola Universitària d’Infermeria i Fisioteràpia Gimbernat (EUG), Universitat Autònoma de Barcelona (UAB), 08174 Barcelona, Spain; amalia.sillero@eug.es

**Keywords:** cystic fibrosis, vascular access devices, ultrasonography, advanced practice nursing, home care services, hospital-based, venous catheterization, peripheral

## Abstract

**Highlights:**

**What are the main findings?**

- An Advanced Practice Nursing-led multidisciplinary care pathway integrated ultrasound-guided vascular assessment, evidence-informed device selection, and coordinated multidisciplinary care in the management of cystic fibrosis.
- The illustrative patient completed prolonged home-based intravenous therapy through an integrated multidisciplinary vascular access pathway.

**What are the implications of the main findings?**

- The pathway may serve as a basis for future evaluation of structured vascular access planning in patients requiring repeated intravenous therapy.
- Advanced Practice Nurses contribute to vascular assessment, care coordination, patient education, and multidisciplinary decision-making within vascular access pathways.

**Abstract:**

**Background**: Patients with cystic fibrosis frequently require repeated courses of intravenous antibiotic therapy, resulting in cumulative vascular injury, progressive depletion of peripheral venous access, and increasing complexity in long-term vascular access management. Integrating structured vascular access planning into multidisciplinary care pathways has become increasingly important for preserving venous capital, optimising vascular access decision-making, and ensuring continuity of care. **Objective**: To describe the development and implementation of an Advanced Practice Nursing-led multidisciplinary vascular access care pathway for patients with cystic fibrosis requiring prolonged intravenous therapy. **Methods**: A practice-based descriptive analysis of a multidisciplinary vascular access pathway was conducted, incorporating ultrasound vascular assessment, structured clinical decision-making, and coordinated care across the Emergency Department, Cystic Fibrosis Unit, Vascular Access and Infusion Team, Hospital Pharmacy Service, and Hospital-at-Home Programme. An illustrative patient example was used to demonstrate pathway implementation in routine clinical practice. **Results**: Pathway implementation illustrated the feasibility of integrating ultrasound-guided vascular assessment, evidence-informed selection of vascular access devices, and coordinated home-based intravenous therapy within a multidisciplinary care model. Ultrasound-guided vascular assessment identified a suitable upper-arm basilic vein despite severe peripheral venous depletion. An Advanced Practice Nurse successfully performed ultrasound-guided midline catheter insertion, achieving first-attempt cannulation and no immediate complications. The illustrative patient completed four weeks of home-based intravenous antibiotic therapy without observed catheter-related infection, thrombosis, occlusion, catheter dysfunction, or unplanned hospital readmissions. The patient reported satisfaction with the treatment experience and the ability to maintain daily activities throughout treatment. **Conclusions**: This study describes and clinically contextualises an APN-led multidisciplinary vascular access care pathway for patients with cystic fibrosis requiring prolonged intravenous therapy. The illustrative patient demonstrated the feasibility of applying the pathway in routine clinical practice. Further prospective multicentre studies are required to evaluate its effectiveness, safety, reproducibility, and broader applicability.

## 1. Introduction

Cystic fibrosis (CF) is a life-limiting autosomal recessive disorder caused by mutations in the CFTR gene, leading to chronic respiratory disease, recurrent pulmonary exacerbations, and progressive multisystem involvement [1]. Despite advances in multidisciplinary care and CFTR modulator therapies, many patients continue to require repeated courses of intravenous (IV) antibiotic therapy throughout their lives [2,3,4]. Consequently, vascular access has become a critical component of long-term disease management, requiring repeated interventions throughout the patient’s lifespan and influencing both current treatment and future opportunities for vascular access.

Recurrent intravenous treatments contribute to cumulative peripheral venous injury, including endothelial damage, fibrosis, venous stenosis, and progressive loss of suitable access sites [5,6]. Because vascular damage often begins in childhood and accumulates throughout life, vascular access should be considered a longitudinal clinical resource requiring proactive preservation and planning across the disease trajectory [5].

Contemporary vascular access frameworks, including Vessel Health and Preservation (VHP), GAVeCeLT, and the Michigan Appropriateness Guide for Intravenous Catheters (MAGIC), emphasise early vascular assessment, appropriate device selection, and strategies to minimise cumulative vascular injury while preserving future access opportunities [7,8,9,10].

Despite these recommendations, short peripheral catheters remain widely used in many clinical settings, particularly for outpatient or home-based intravenous therapy. However, their limited dwell time and high failure rates are associated with repeated venepunctures, increased patient discomfort, and cumulative vascular trauma [11]. Midline catheters represent an intermediate option between short peripheral and central venous access devices and may provide reliable vascular access for therapies lasting several weeks while avoiding central venous cannulation [8]. Their use should be guided by careful vascular assessment and integrated within long-term vascular preservation strategies [9,10].

Ultrasound-guided vascular access is recommended in patients with difficult intravenous access because it improves vessel visualisation, facilitates appropriate vessel selection, and increases first-attempt success rates [12,13]. The incorporation of ultrasound into vascular access practice has expanded the role of Advanced Practice Nurses, who contribute to vascular assessment, device selection, insertion procedures, patient education, and care coordination. Previous studies have shown that APN-led vascular access services may improve clinical outcomes and patient experience [7,14,15].

However, despite advances in vascular access technologies and professional competencies, management is still often treated as an isolated, reactive technical procedure rather than as part of a structured, multidisciplinary care pathway. Key elements such as anticipatory vascular planning, coordination across services, and integration with home-based treatment models remain underdeveloped in routine practice and underrepresented in the literature [6].

Despite increasing recognition of vessel preservation principles and evidence-based vascular access practices, the literature describing how these components can be integrated into structured APN-led multidisciplinary care pathways for patients with cystic fibrosis is limited.

Therefore, this study aimed to describe the development and implementation of an Advanced Practice Nursing-led multidisciplinary vascular access care pathway for patients with cystic fibrosis requiring prolonged intravenous therapy.

## 2. Materials and Methods

### 2.1. Study Design

This study presents a practice-based descriptive analysis of an Advanced Practice Nursing-led multidisciplinary vascular access pathway in cystic fibrosis. It was informed by principles of complex clinical interventions and care pathway development [16], and described and clinically contextualised how structured vascular assessment, device selection, and coordinated care were integrated into routine clinical practice in accordance with venous capital preservation principles.

Accordingly, the unit of analysis was the multidisciplinary care pathway rather than the individual patient. The clinical application presented served solely to illustrate the pathway’s implementation in routine clinical practice.

A practice-based descriptive approach was adopted because the objective was to describe the implementation of a clinical pathway rather than to establish causal relationships or generate generalisable evidence. The illustrative patient was included exclusively to demonstrate how the pathway operates in routine clinical practice and to facilitate understanding of its implementation within a real clinical context. Therefore, the patient example should not be interpreted as evidence of intervention effectiveness or pathway performance.

### 2.2. Setting and Clinical Context

The study was conducted in a tertiary university hospital with an established multidisciplinary care structure. The Cystic Fibrosis Unit is one of the largest adult cystic fibrosis referral units in Spain and has followed a cohort of 349 adult patients with cystic fibrosis over recent decades [17]. The pathway involved the Emergency Department, Cystic Fibrosis Unit, Vascular Access and Infusion Team (VAIT), Hospital Pharmacy Service, and a Hospital-at-Home Programme.

These services are integrated within an integrated care framework designed to support patients requiring prolonged intravenous therapy and to coordinate clinical decision-making and continuity of care across hospital and home settings. The Vascular Access and Infusion Team plays a central role in coordinating vascular assessment, device selection, and procedural management within this pathway.

The pathway had been implemented in routine clinical practice prior to the preparation of this manuscript and was already being used as part of the institutional approach to vascular access management in patients requiring prolonged intravenous therapy.

### 2.3. Reporting Framework

This study was reported following established reporting frameworks for care pathway descriptions and complex clinical interventions. The description of the care pathway was guided by:

- TIDieR (Template for Intervention Description and Replication), to provide a detailed description of intervention components, delivery, and implementation context [18]. The TIDieR checklist is provided as Supplementary Materials (Supplementary Material S1);
- The European Pathway Association (EPA) framework, which defines integrated care pathways as structured multidisciplinary plans that support coordination of care across settings [19].

These reporting frameworks informed the description of pathway components, roles, and decision-making processes, and facilitated transparent reporting of the intervention.

### 2.4. Data Sources and Analysis

Data were obtained retrospectively from electronic health records, clinical documentation, and patient-reported outcome data. Data were extracted from clinical records and reviewed by the authors. The analysis focused on clinical, procedural, organisational, and patient-reported variables relevant to vascular access management.

To illustrate the implementation of the pathway in routine clinical practice, a patient who had undergone all stages of the multidisciplinary pathway and presented complex vascular access needs was purposively selected as an illustrative example. Clinical variables included demographic characteristics, diagnosis, history of vascular access, and current treatment requirements. Vascular assessment was performed using ultrasound to evaluate vessel diameter, depth, patency, compressibility, and anatomical trajectory.

Vessel selection was guided by structured clinical criteria and established principles such as the Zone Insertion Method (ZIM), prioritising upper-arm veins to optimise catheter performance and minimise mechanical complications [20]. The patient was identified as presenting with difficult intravenous access based on clinical and ultrasound findings. Although a formal A-DIVA score was not systematically applied, recognised predictors were present, including severe venous depletion, multiple previous vascular access procedures, and absence of visible or palpable peripheral veins [21]. Ultrasound-guided vascular access was therefore performed prior to any insertion attempt in accordance with current recommendations [12].

Device selection was guided by a structured decision-making process that integrated treatment duration, medication characteristics, vascular anatomy, and the long-term preservation of venous capital. Concepts related to vessel health and preservation guided the selection of the most appropriate vascular access device within the clinical context [7].

Procedural variables included insertion characteristics, number of cannulation attempts, catheter dwell time, and catheter-related complications. Organisational variables focused on multidisciplinary coordination across services.

Patient-reported outcomes were collected at the end of treatment through a structured post-treatment assessment addressing treatment experience, perceived comfort, impact on daily activities, and satisfaction with care. Data collection covered the period from vascular access assessment and catheter insertion through completion of the four-week intravenous treatment and follow-up process.

Data were analysed descriptively to characterise pathway implementation and to provide an integrated description of clinical, organisational, procedural, and patient-centred outcomes.

No statistical analysis was performed because the study was descriptive in nature and used a single illustrative patient example.

### 2.5. Description of the Care Pathway

The vascular access care pathway was structured as a coordinated, multidisciplinary process integrating clinical assessment, vascular decision-making, technical intervention, and continuity of care across hospital and home settings.

The pathway is routinely used in clinical practice for patients requiring prolonged intravenous therapy and complex vascular access management. For the purpose of this manuscript, a single patient example is presented solely to illustrate its implementation in routine practice.

The pathway is intended for patients requiring prolonged intravenous therapy and presenting anticipated or established difficulties in vascular access management.

The pathway involved Advanced Practice Nurses with advanced training and expertise in ultrasound-guided vascular access, together with physicians and nurses from the Emergency Department, Cystic Fibrosis Unit, Hospital Pharmacy Service, Vascular Access and Infusion Team, and Hospital-at-Home Programme.

The pathway consisted of the following components:

- Initial clinical evaluation:

The process starts in the Emergency Department, where the indication for intravenous therapy is established. In patients with suspected or confirmed difficult intravenous access, early referral to the Vascular Access and Infusion Team (VAIT) is recommended to avoid repeated attempts at cannulation.

- APN-led ultrasound vascular assessment:

A comprehensive ultrasound assessment is performed by an Advanced Practice Nurse (APN), including evaluation of vessel diameter, depth, patency, compressibility, and anatomical trajectory, to identify suitable veins and support subsequent decision-making.

- Structured clinical decision-making:

Device selection was guided by a structured approach integrating treatment duration, medication characteristics, vascular anatomy, and long-term preservation of venous capital, in accordance with established vascular access frameworks.

- Ultrasound-guided catheter insertion:

Midline catheter insertion is performed under ultrasound guidance using aseptic technique and a modified Seldinger approach to maximise first-attempt success and minimise procedural complications.

- Coordinated transition to home-based care:

Following device placement, discharge planning was coordinated through structured multidisciplinary follow-up, including patient education, pharmacy validation, and activation of the Hospital-at-Home Programme.

- Home-based intravenous therapy and follow-up:

Intravenous treatment is continued under structured clinical supervision, with regular monitoring of catheter function, insertion site condition, and early detection of complications.

- End-of-therapy evaluation:

At treatment completion, clinical outcomes, catheter-related events, and patient-reported experience are assessed to support evaluation and ongoing refinement of the pathway.

**Figure 1:**
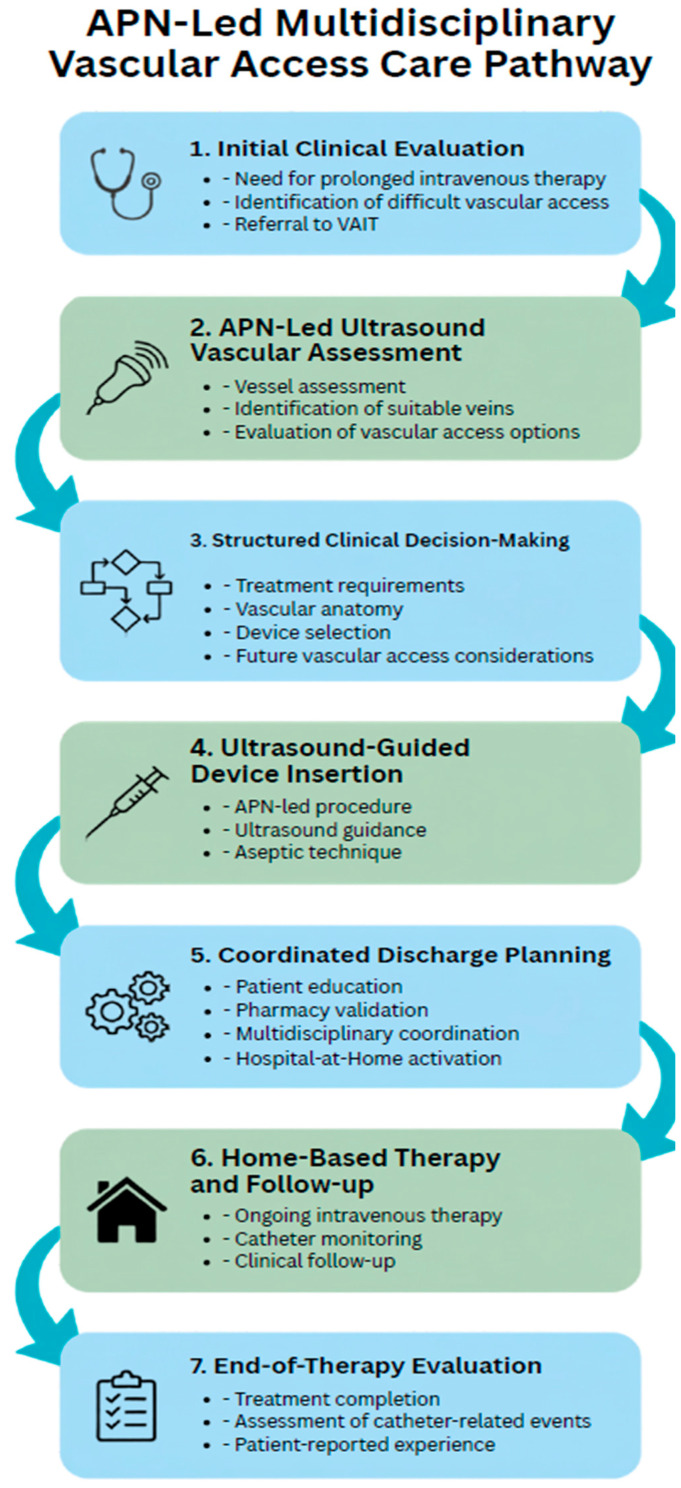
APN-Led Multidisciplinary Vascular Access Care Pathway. Note: This figure illustrates the structure of an APN-led multidisciplinary vascular access care pathway integrating vascular assessment, clinical decision-making, device selection, patient education, and continuity of care across hospital and home settings. Generated with the assistance of artificial intelligence and reviewed by the authors.

The overall structure, sequence, and clinical timeline of the vascular access pathway are illustrated in Figure 1.

### 2.6. Treatment and Follow-Up

Intravenous antibiotic therapy was administered in accordance with institutional outpatient parenteral antimicrobial therapy (OPAT) protocols and hospital pharmacy recommendations. Medication preparation, storage, and administration followed established procedures to ensure physicochemical stability and safe delivery in the home-care setting [22]. Treatment regimens were adapted to the patient’s clinical condition and microbiological profile in coordination with the Cystic Fibrosis Unit.

Clinical follow-up was conducted according to the predefined multidisciplinary pathway through the Hospital-at-Home Programme in collaboration with the Vascular Access and Infusion Team (VAIT) and the Cystic Fibrosis Unit. Monitoring included regular assessment of catheter function and insertion site condition, as well as early detection of potential complications such as infection, thrombosis, or occlusion. Catheter-related complications were monitored through routine clinical follow-up, including evaluation of catheter function, insertion site condition, signs of infection, evidence of thrombosis, and the occurrence of catheter occlusion, infiltration, dislodgement, or unplanned catheter removal.

In addition, patient and family education on catheter care, treatment adherence, and recognition of warning signs was reinforced throughout the follow-up period to facilitate safe self-management and continuity of care in the home setting.

The duration of the intervention corresponded to the planned four-week intravenous antibiotic treatment.

### 2.7. Ethical Considerations

The study was performed in accordance with the principles of the Declaration of Helsinki and approved by the Clinical Research Ethics Committee of the corresponding institution (approval code PR(AG)418/2023).

Written informed consent was obtained for the use of anonymised clinical information included in the description of the care pathway. All data were anonymised prior to analysis, in compliance with Regulation (EU) 2016/679 (General Data Protection Regulation, GDPR) and applicable national data protection legislation [23].

## 3. Results

### 3.1. Multidisciplinary Care Pathway

A structured multidisciplinary vascular access pathway was successfully implemented in routine clinical practice, involving the Emergency Department, Cystic Fibrosis Unit, Vascular Access and Infusion Team, Hospital Pharmacy Service, and Hospital-at-Home Programme.

The multidisciplinary care pathway integrates clinical evaluation, ultrasound-guided vascular assessment, structured device selection, procedural expertise, and coordinated continuity of care within a multidisciplinary care model. Early vascular assessment, evidence-informed clinical decision-making, and coordinated multidisciplinary care represent key components of the pathway across hospital and home settings.

Early ultrasound-guided vascular assessment, evidence-informed clinical decision-making, and Advanced Practice Nursing leadership represented the core components of the pathway, supporting both procedural management and organisational coordination. Integration of hospital-based and home-based care further facilitated treatment delivery in the home setting while ensuring ongoing clinical oversight. The pathway illustrated how structured vascular assessment, evidence-informed decision-making, and multidisciplinary coordination into routine clinical practice.

### 3.2. Vascular Assessment and Decision-Making

Vascular assessment revealed significant depletion of peripheral venous access, consistent with the cumulative impact of repeated intravenous therapies. Clinical examination showed limited visibility and palpability of superficial veins, indicating a high likelihood of difficult intravenous access.

Ultrasound evaluation was performed prior to any insertion attempt, allowing detailed assessment of vessel diameter, depth, patency, compressibility, and anatomical trajectory. Multiple venous segments showed signs of fibrosis and reduced suitability for catheterisation, considerably limiting potential access sites.

A patent basilic vein in the upper arm was identified as the most appropriate access site, demonstrating adequate calibre, favourable depth, full compressibility, and absence of thrombosis. The catheter-to-vein ratio (CVR) was maintained within recommended limits.

Based on clinical and ultrasound findings, the patient was considered to have difficult intravenous access, with recognised predictors including severe venous depletion, multiple previous vascular access procedures, and absence of visible or palpable peripheral veins.

Device selection was guided by integration of treatment duration, medication characteristics, vascular anatomy, and long-term venous preservation. Short peripheral catheters were considered unsuitable due to the high likelihood of early failure, and the use of a peripherally inserted central catheter (PICC) was deferred to preserve central venous access.

A midline catheter was selected as the most appropriate option to balance treatment requirements with preservation of venous capital.

### 3.3. Application of the Care Pathway in Clinical Practice

To illustrate pathway implementation, the care model was applied in a patient requiring prolonged intravenous antibiotic therapy for a pulmonary exacerbation of cystic fibrosis. Following initial evaluation in the Emergency Department, targeted antimicrobial therapy was initiated based on clinical and microbiological findings. Baseline clinical characteristics and relevant medical history are summarised in Table 1.

Given the complexity of vascular access and the identified limitations in peripheral venous availability, early involvement of the Vascular Access and Infusion Team was prioritised. Ultrasound-guided assessment enabled identification of a suitable access site and supported timely decision-making.

Midline catheter insertion was performed by an Advanced Practice Nurse using a modified Seldinger technique under sterile conditions. Cannulation was achieved on the first attempt without immediate complications. Continuous ultrasound guidance ensured accurate vessel targeting and appropriate catheter positioning.

Following device placement, a coordinated multidisciplinary care plan was established, involving the Emergency Department, Cystic Fibrosis Unit, Vascular Access Team, Hospital Pharmacy Service, and Hospital-at-Home Programme. This approach enabled early transition from hospital-based care to home-based intravenous therapy.

The patient received structured education on catheter care, infection prevention, and recognition of potential complications to support safe self-management. Intravenous treatment was continued using portable infusion systems in accordance with outpatient parenteral antimicrobial therapy protocols.

Clinical follow-up was conducted through the Hospital-at-Home Programme, with regular assessments of catheter function and early detection of potential complications, ensuring continuity of care and adherence to treatment.

The Advanced Practice Nurse played a central role throughout the pathway, contributing to vascular assessment, device selection, procedural management, patient education, and coordination across services. These contributions are summarised in Table 2.

### 3.4. Clinical and Patient-Reported Findings

Pathway implementation enabled completion of the planned four-week course of intravenous antibiotic therapy through the midline catheter. Procedural characteristics and ultrasound findings are summarised in Table 3.

The catheter remained fully functional throughout the treatment period and did not require replacement. No catheter-related complications were documented during the observation period, including infection, thrombosis, occlusion, infiltration, dislodgement or unplanned catheter removal. Catheter patency was maintained without interruption of therapy. No unplanned hospital readmissions were documented during treatment, and clinical and radiological follow-up confirmed resolution of the pulmonary exacerbation.

From a patient-centred perspective, the patient described satisfaction with the treatment experience, a reduced need for repeated venepuncture, perceived comfort during therapy, and the ability to maintain daily activities throughout home-based treatment. These observations were obtained through the structured post-treatment assessment conducted at the end of therapy and are presented descriptively rather than as validated patient-reported outcome measures.

## 4. Discussion

This study presents a multidisciplinary, Advanced Practice Nursing-led vascular access care pathway in cystic fibrosis, illustrating how structured vascular assessment, evidence-informed decision-making, and coordinated multidisciplinary care can be integrated into routine clinical practice for patients requiring repeated intravenous therapy.

In cystic fibrosis, repeated intravenous treatments contribute to progressive venous depletion, thereby increasing the complexity of vascular access. In this illustrative patient, severe peripheral venous depletion, the absence of visible or palpable veins, and multiple previous vascular access procedures were consistent with recognised predictors of difficult intravenous access [21]. Within this context, ultrasound-guided vascular assessment played a central role in identifying a suitable access site and facilitating evidence-informed decision-making. Previous studies have demonstrated that ultrasound guidance improves first-attempt success rates and reduces insertion-related complications, particularly in patients with difficult vascular access [24,25].

Importantly, the principal contribution of this study lies not only in the selection of an appropriate vascular access device but also in the implementation of a structured multidisciplinary care pathway integrating ultrasound-guided vascular assessment, evidence-informed device selection, and coordinated continuity of care. From an implementation perspective, the pathway illustrates how evidence-based vascular access principles can be translated into routine clinical practice through multidisciplinary collaboration.

Beyond achieving vascular access for the immediate episode of care, preserving future vascular access options has become an important consideration in clinical decision-making. Contemporary vascular access frameworks emphasise that device selection should consider not only short-term treatment needs but also anticipated future vascular access requirements. This perspective is particularly relevant in cystic fibrosis, where lifelong exposure to intravenous therapies may progressively compromise both peripheral and central venous access, making proactive vascular access planning an essential component of comprehensive disease management [7,26].

Within the presented multidisciplinary care pathway, midline catheters represented an intermediate option between short peripheral and central venous access devices, allowing prolonged intravenous therapy while avoiding the risks associated with central catheterisation.

Current evidence supports the use of midline catheters in selected clinical scenarios, with systematic reviews demonstrating favourable safety profiles when appropriate patient selection and monitoring are applied [27,28]. Accordingly, appropriate device selection should be regarded as one component of a broader multidisciplinary vascular access strategy rather than an isolated technical decision.

Although no catheter-related complications were observed during the treatment period, prolonged use of midline catheters requires careful patient selection and ongoing monitoring. Factors such as treatment duration, vascular characteristics, medication properties, and individual clinical complexity should be considered when evaluating the appropriateness of a midline catheter and ensuring safe device management [28].

In the present case, the catheter tip location was considered appropriate according to institutional practice and the selected vascular access strategy. However, considerations regarding catheter tip location, device choice, and monitoring practices may vary across clinical settings and should be individualised according to patient characteristics and treatment requirements [10].

Recent comparative studies have reported conflicting findings regarding the safety and effectiveness of midline catheters versus peripherally inserted central catheters (PICCs), underscoring that no single vascular access device is universally appropriate across all clinical situations [29,30]. While some studies have reported higher complication rates associated with midline catheters [29], others have identified lower rates of major complications and catheter-related bloodstream infections in outpatient settings [30]. These discrepancies suggest that device selection should not rely solely on catheter type, but rather on individual patient characteristics, vascular anatomy, treatment duration, and the broader clinical context.

Within the multidisciplinary pathway presented, treatment was completed without documented catheter-related complications while following principles of venous capital preservation. Although these findings cannot be generalised, they illustrate how individualised vascular access planning, supported by ultrasound-guided assessment and structured decision-making, may support vascular access management in patients with complex vascular access needs. From an implementation perspective, the favourable outcome observed should not be attributed solely to the vascular access device but rather interpreted within the context of the multidisciplinary pathway.

Beyond the technical aspects of vascular access, the present study highlights the contribution of Advanced Practice Nurses within multidisciplinary care pathways. In the present pathway, the APN role extended beyond catheter insertion to include vascular assessment, clinical decision-making, patient education, and coordination across services, facilitating communication between hospital- and community-based teams.

Previous evidence has shown that advanced practice nursing roles are associated with improved care coordination, healthcare efficiency, and positive clinical outcomes [31,32,33,34]. Although the present study was not designed to evaluate these outcomes, it illustrates how APN leadership may support the implementation of a multidisciplinary vascular access pathway in routine clinical practice.

Structured vascular assessment, evidence-informed clinical decision-making, multidisciplinary collaboration, APN leadership, and longitudinal follow-up were integrated within the pathway and formed part of the multidisciplinary pathway during treatment. This observation aligns with evidence supporting integrated care models for patients with chronic conditions. However, the present study was not designed to evaluate the impact of the pathway on clinical outcomes or healthcare utilisation [35].

The integration of hospital-based and home-based care through outpatient parenteral antimicrobial therapy constitutes a key component of the multidisciplinary pathway. Hospital-at-Home programmes have demonstrated safety and effectiveness in managing patients with chronic conditions while reducing hospital stays [35]. Within the present pathway, this model was used to provide continuity of care across healthcare settings while maintaining appropriate clinical oversight and allowing treatment delivery in a less restrictive environment.

From a patient-reported perspective, the illustrative patient reported satisfaction with the treatment experience and the ability to maintain daily activities throughout treatment. These observations provide useful insights into the patient’s experience but should be interpreted descriptively and cannot be considered evidence of improved patient-centred outcomes. Nevertheless, they highlight the importance of considering patient experience alongside procedural and clinical aspects when evaluating vascular access pathways. This perspective is consistent with person-centred care frameworks, which recognise patient experience, autonomy, and reduced treatment burden as important dimensions of healthcare quality [36,37].

Taken together, this manuscript illustrates how structured vascular access pathways may integrate ultrasound-guided assessment, evidence-informed clinical decision-making, Advanced Practice Nursing leadership, and multidisciplinary coordination within routine clinical practice. Future research should evaluate such pathways in larger populations and across different healthcare settings, focusing on vascular access preservation, complication rates, patient experience, treatment adherence, and healthcare utilisation. These multidisciplinary care pathways may also warrant future evaluation in other chronic conditions requiring repeated intravenous therapy, including oncology, nephrology, and infectious diseases.

Future implementation studies should incorporate APN-sensitive outcomes, healthcare delivery indicators, and health service utilisation measures to evaluate the reproducibility, scalability, and sustainability of multidisciplinary vascular access pathways across different healthcare settings.

### Strengths and Limitations

This study has several limitations that should be acknowledged. First, this study was designed to describe and clinically contextualise the implementation of a multidisciplinary vascular access pathway in routine clinical practice rather than to evaluate its effectiveness. Consequently, the findings should be interpreted as illustrating implementation and demonstrating the feasibility and clinical applicability of the proposed pathway within the clinical context described, rather than providing definitive evidence of efficacy or generalisability.

Second, the descriptive and observational design, together with the absence of comparative analyses involving alternative vascular access strategies, precludes conclusions regarding the comparative effectiveness and safety of the proposed pathway. Furthermore, a validated assessment tool for difficult intravenous access, such as the A-DIVA scale, was not systematically applied, and long-term vascular outcomes and health economic analyses were beyond the scope of this study. In addition, venous capital preservation was not formally measured and therefore should not be interpreted as a demonstrated outcome of the pathway. Furthermore, systematic surveillance for asymptomatic thrombosis was not performed. Therefore, the absence of clinically evident thrombosis should be interpreted with caution.

The purposive selection of a single illustrative patient may have introduced selection bias, as the case was chosen to demonstrate pathway implementation in routine clinical practice. Observer bias cannot be excluded because data were extracted from clinical documentation and reviewed by the authors. The use of a single illustrative patient also limits the applicability of the observations to other clinical settings. Despite these limitations, the study has important strengths. Rather than evaluating the performance of a specific vascular access device, it provides a clinically grounded description of an Advanced Practice Nursing-led multidisciplinary pathway for vascular access management implemented in routine clinical practice. By integrating ultrasound-guided vascular assessment, evidence-informed clinical decision-making, coordinated multidisciplinary care, and patient experience, the proposed pathway offers a structured description of multidisciplinary vascular access management that may inform future implementation studies.

## 5. Conclusions

This study describes and clinically contextualises an Advanced Practice Nursing (APN)-led multidisciplinary vascular access pathway supported by ultrasound-guided vascular assessment for patients with cystic fibrosis requiring prolonged intravenous therapy. The findings illustrate how structured vascular assessment, evidence-informed clinical decision-making, and coordinated multidisciplinary care can be integrated into routine practice to support vascular access decision-making, to support vascular access decision-making in accordance with principles of venous capital preservation.

The proposed pathway reflects a shift from considering vascular access as an isolated technical procedure to recognising it as a longitudinal component of chronic disease management. Within this model, structured ultrasound-guided vascular assessment, appropriate device selection, APN leadership, and multidisciplinary collaboration are integrated to provide continuity of care and vascular access planning.

Although further prospective multicentre studies are required to evaluate its effectiveness, the present study illustrates how the pathway was implemented in routine clinical practice through a single illustrative patient. However, the present study was not designed to evaluate effectiveness, safety, reproducibility, or transferability.

Future prospective multicentre studies are needed to evaluate the implementation, effectiveness, sustainability, and healthcare delivery outcomes of similar multidisciplinary pathways using predefined clinical, patient-reported, implementation, and economic outcomes before broader implementation or transferability to other healthcare settings can be recommended.

## Figures and Tables

**Table 1:** Clinical characteristics relevant to pathway implementation.

Variable	Finding
Age	Early thirties
Sex	Female
Primary diagnosis	Cystic fibrosis
Lung transplantation history	Bilateral lung transplantation at age 13
Previous acute rejection episode	Yes
Chronic renal insufficiency	Yes
Exocrine pancreatic insufficiency	Yes
Osteoporosis	Yes
Documented drug allergies	Metamizole, ciprofloxacin, valganciclovir, levofloxacin
Previous IV antibiotic courses	>12
Peripheral venous status	Severe venous depletion with visible scarring
Planned treatment duration	Four weeks
Prescribed antibiotic	Ceftazidime–avibactam

**Table 2:** Contribution of the Advanced Practice Nurse throughout the care pathway.

Phase	APN Contribution
Assessment	Ultrasound vascular assessment
Planning	Device selection and risk-benefit analysis
Procedure	Ultrasound-guided insertion
Education	Patient and family education
Coordination	Liaison with CF Unit, Pharmacy and Hospital-at-Home
Follow-up	Surveillance and complication monitoring
Overall contribution	Support for treatment completion and consideration of future vascular access needs

**Table 3:** Procedural and vascular assessment findings during pathway implementation.

Variable	Finding
Selected vessel	Right basilic vein
Vessel diameter	3.2 mm
Vessel depth	1.2 cm
Vessel patency	Preserved
Evidence of thrombosis	Not detected
Device type	Midline catheter
Catheter gauge	20 G
Catheter length	10 cm
Insertion technique	Ultrasound-guided modified Seldinger
Insertion attempts	1
Procedure duration	Approximately 10 min
Tip position	Proximal axillary vein
Immediate complications	None

## Data Availability

The data presented in this study are not publicly available due to privacy and ethical restrictions but are available from the corresponding author on reasonable request.

## Supplementary Materials

The following supporting information can be downloaded at: https://www.mdpi.com/article/10.3390/healthcare14152414/s1, Supplementary Material S1: TIDieR Checklist.

## Abbreviations

The following abbreviations are used in this manuscript:

A-DIVA	Adult Difficult Intravenous Access Scale
APN	Advanced Practice Nurse
CF	Cystic Fibrosis
CFTR	Cystic Fibrosis Transmembrane Conductance Regulator
CVR	Catheter-to-Vein Ratio
DIVA	Difficult Intravenous Access
ED	Emergency Department
GAVeCeLT	Gruppo Accessi Venosi Centrali a Lungo Termine
IV	Intravenous
MAGIC	Michigan Appropriateness Guide for Intravenous Catheters
OPAT	Outpatient Parenteral Antimicrobial Therapy
PICC	Peripherally Inserted Central Catheter
VAIT	Vascular Access and Infusion Team
VHP	Vessel Health and Preservation
ZIM	Zone Insertion Method
