# Supplementary material for: Multidisciplinary Advanced Practice Nursing-Led Vascular Access Care Pathway in Patients with Cystic Fibrosis: A Practice-Based Descriptive Analysis
Source: Healthcare (Basel). 2026 Aug 5;14(15):2414. doi: 10.3390/healthcare14152414 (PMC13464999; doi:10.3390/healthcare14152414)
Supplement: Supplementary file 1 [file healthcare-14-02414-s001.zip › healthcare-4466456-supplementary.pdf]

The TIDieR (Template for Intervention Description and Replication) Checklist*:

Information to include when describing an intervention and the location of the information

Item number		Primary paper (page or appendix number)	Other † (details)
	**BRIEF NAME**		
**1.**	Provide the name or a phrase that describes the intervention.	Methods 2.1.- 2.2.	APN-led multidisciplinary vascular access care pathway in cystic fibrosis
	**WHY**		
**2.**	Describe any rationale, theory, or goal of the elements essential to the intervention.	Introduction and Methods 2.1.	
	**WHAT**		
**3.**	Materials: Describe any physical or informational materials used in the intervention, including those provided to participants or used in intervention delivery or in training of intervention providers. Provide information on where the materials can be accessed (e.g. online appendix, URL).	Methods 2.4 and 2.6	Ultrasound equipment for vascular assessment, midline catheter insertion kits, and outpatient parenteral antimicrobial therapy (OPAT) systems, including portable infusion devices. Educational materials were provided to the patient and family to

			support catheter care, treatment adherence, and recognition of complications.
**4.**	Procedures: Describe each of the procedures, activities, and/or processes used in the intervention, including any enabling or support activities.	Methods 2.5	The intervention consisted of a structured vascular access care pathway including: (a) initial evaluation in the Emergency Department, (b) APN-led ultrasound vascular assessment, (c) structured device selection, (d) ultrasound-guided midline catheter insertion, (e) coordinated discharge planning, (f) home-based intravenous therapy under the Hospital-at-Home Programme, and (g) end-of-therapy evaluation
	**WHO PROVIDED**		
**5.**	For each category of intervention provider (e.g. psychologist, nursing assistant), describe their expertise, background and any specific training given.	Methods 2.2 and 2.5	The intervention was primarily delivered by an Advanced Practice Nurse (APN) specialised in vascular access, who performed vascular assessment, device selection, catheter insertion, patient education, and care coordination. The intervention was supported by a multidisciplinary team including emergency physicians, cystic fibrosis specialists, hospital pharmacists, and Hospital-at-Home professionals.
	**HOW**		

**6.**	Describe the modes of delivery (e.g. face-to-face or by some other mechanism, such as internet or telephone) of the intervention and whether it was provided individually or in a group.	Methods 2.2 and 2.6	The intervention was delivered through a combination of hospital-based and home-based care, including face-to-face clinical assessment, ultrasound-guided procedures, and coordinated follow-up via the Hospital-at-Home Programme.
	**WHERE**		
**7.**	Describe the type(s) of location(s) where the intervention occurred, including any necessary infrastructure or relevant features.	Methods 2.2.	The intervention was carried out in a tertiary university hospital setting and extended to the patient's home through a structured Hospital-at-Home Programme.
	**WHEN and HOW MUCH**		
**8.**	Describe the number of times the intervention was delivered and over what period of time including the number of sessions, their schedule, and their duration, intensity or dose.	Methods 2.6 and Results 3.4	The intervention was delivered over a four-week period corresponding to the duration of intravenous antibiotic therapy, including initial hospital assessment, catheter insertion, and continuous home-based treatment with regular follow-up.
	**TAILORING**		
**9.**	If the intervention was planned to be personalised, titrated or adapted, then describe what, why, when, and how.	Methods 2.6 and 2.5	The intervention was individualised based on vascular assessment findings, patient clinical condition, treatment

			requirements, and future vascular access considerations.
	**MODIFICATIONS**		
**10.‡**	If the intervention was modified during the course of the study, describe the changes (what, why, when, and how).	N/A	
	**HOW WELL**		
**11.**	Planned: If intervention adherence or fidelity was assessed, describe how and by whom, and if any strategies were used to maintain or improve fidelity, describe them.	Methods 2.5	No formal assessment of intervention fidelity was planned; however, standardised procedures and multidisciplinary coordination were applied to support consistent implementation of the pathway.
**12.‡**	Actual: If intervention adherence or fidelity was assessed, describe the extent to which the intervention was delivered as planned.	Results 3.3-3.4	The pathway components were delivered as planned during the illustrative implementation described, including ultrasound-guided assessment, device selection, catheter insertion, multidisciplinary coordination, and completion of intravenous therapy.

** **Authors** - use N/A if an item is not applicable for the intervention being described. **Reviewers** – use '?' if information about the element is not reported/not sufficiently reported.

† If the information is not provided in the primary paper, give details of where this information is available. This may include locations such as a published protocol or other published papers (provide citation details) or a website (provide the URL).

‡ If completing the TIDieR checklist for a protocol, these items are not relevant to the protocol and cannot be described until the study is complete.

* We strongly recommend using this checklist in conjunction with the TIDieR guide (see *BMJ* 2014;348:g1687) which contains an explanation and elaboration for each item.

* The focus of TIDieR is on reporting details of the intervention elements (and where relevant, comparison elements) of a study. Other elements and methodological features of

studies are covered by other reporting statements and checklists and have not been duplicated as part of the TIDieR checklist. When a **randomised trial** is being reported, the TIDieR checklist should be used in conjunction with the CONSORT statement (see www.consort-statement.org) as an extension of **Item 5 of the CONSORT 2010 Statement.** When a **clinical trial protocol** is being reported, the TIDieR checklist should be used in conjunction with the SPIRIT statement as an extension of **Item 11 of the SPIRIT 2013 Statement** (see www.spirit-statement.org). For alternate study designs, TIDieR can be used in conjunction with the appropriate checklist for that study design (see www.equator-network.org).
